# The expression of genes in different sites of gut tract regulates the meat quality of semitendinosus muscle in sheep and goats

**DOI:** 10.3389/fvets.2025.1687258

**Published:** 2025-10-29

**Authors:** Binlong Chen, Zhiying Huang, Zhongkun Cai, Siyu Li, Guangwen Yan, Weihua Chang, Yi Zhang, Shizhong Yang

**Affiliations:** ^1^College of Animal Science, Xichang University, Xichang, China; ^2^College of Animal Sciences, Shanxi Agricultural University, Taigu, China; ^3^School of Pharmacy, Chengdu University, Chengdu, China; ^4^Liangshan Academy of Agricultural Sciences, Xichang, China

**Keywords:** gut sites, gene expression, semitendinosus muscle, meat quality, sheep and goat

## Abstract

**Introduction:**

For small ruminants, meat quality—an economically significant characteristic—results from the combined effects of genetic, dietary, and physiological elements. However, the contribution of gastrointestinal (GI) tract gene expression to meat quality remains unclear.

**Methods:**

Here, we performed bulk RNA-seq on 130 samples from Liangshan Black Sheep and Meigu Black Goats, including 10 GI tract segments and semitendinosus muscle, integrating these data with measurements of amino acid composition, fatty acid profiles, and volatile flavor compounds.

**Results and discussion:**

We found distinct, segment-specific transcriptional programs across the GI tract, with major functional shifts at the rumen–reticulum, omasum–abomasum, and abomasum–duodenum transitions. In the ileum and jejunum, genes involved in lipid metabolism showed links to fatty acid profiles, whereas genes governing amino acid metabolism in the small intestine were connected to the amino acid composition of muscle. Cecum- and colon-enriched genes were linked to flavor precursor biosynthesis. Species-specific differences revealed that sheep muscle contained higher levels of key amino acids (Asp, Glu, Hyp, Cys, Tyr), whereas goats showed higher α-linolenic acid and other polyunsaturated fatty acids. This work establishes a gut–muscle transcriptomic axis in small ruminants, identifying candidate genes (e.g., *GKN2*, *APOC3*, *AQP5*) and pathways that may be involved in regulating amino acid, fatty acid, and flavor traits of meat quality.

## 1 Introduction

As the living standards of people around the world and in China continue to rise, the demand for mutton is also increasing. At present, worldwide red meat output surpasses 337 million tons, with mutton accounting for around 1.82% (approximately 6.14 million tons) and lamb for 2.93% (about 9.88 million tons). According to the latest research, the global mutton market size is expected to reach about 50 billion dollars by 2025 and 75.55 billion dollars by 2030. However, as consumers pay more attention to healthy eating, the demand for low-fat, high-protein mutton is increasing, and high-quality mutton has higher economic value in the market (1). Moreover, high-quality mutton is not only superior in taste but also richer in essential amino acids and unsaturated fatty acids, making it more beneficial for health (2). In China, Meigu Black Goats and Liangshan Black Sheep are two local high-quality breeds, each with unique breeding environments and meat characteristics (3). Meigu Black Goats are well known for having soft, delicate meat, a mild odor, and a high level of protein, and are primarily found in Meigu County, Liangshan Prefecture, Sichuan Province. Their breeding environment is primarily mountainous, suitable for grazing (4). Liangshan Black Sheep, in contrast, are noted for having succulent and juicy flesh with only a mild odor. They are mainly found in the cold highland areas of Liangshan Prefecture and are characterized by their ability to thrive on coarse feed and strong adaptability (4).

The characteristics of mutton are shaped by multiple factors, including heredity, animal age, diet, and sex (5). In practice, livestock meat quality is shaped not only by genetic background, feeding practices, and nutrient intake but also by aspects such as gene activity, the chemical makeup of the meat, and how tissues are proportioned (6–8). For example, the polymorphism of *PRKAG3* affects intramuscular fat deposition and carcass fat deposition levels in goats (9). High-protein feed can significantly increase the abdominal fat mass in lambs (10). Outdoor grazing increases the movement and metabolic activity of sheep, promoting fat deposition in muscles (11).

Moreover, the digestive system—consisting of the stomach along with the small and large intestines—has a crucial function in breaking down food, taking up nutrients, and regulating metabolic processes (12, 13). Host metabolism is regulated by multiple factors, among which gastrointestinal gene expression plays a central role (12, 14, 15). Transcriptional activities in different gut regions influence nutrient absorption and metabolic processes, which in turn affect muscle development and meat quality traits such as tenderness, juiciness, and flavor (16–20). Moreover, the host’s transcriptional activities are crucial for muscle formation. For example, differential activity of genes governing oxidative muscle fibers (e.g., *MYL2* and *HOXA/C/D*) can alter muscle contraction characteristics, energy utilization, and the deposition of flavor compounds (21). Recently, advances in high-throughput sequencing have greatly promoted intestinal transcriptomics research in pigs, cattle, chickens, and other animals. For example, Yu et al. found that genes such as *KRT85*, *CLDN1*, and *S100A12* may promote intestinal inflammation in pigs by studying the expression of inflammation-related genes in the colon tissue of pigs under different heat stress conditions (22). In addition, Park et al. used RNA-seq to generate a transcriptomic map of cattle rumen tissue and observed substantial variations in gene expression across different growth stages, affecting multiple functional modules such as immune response, steroid metabolism, and protein synthesis (23). In summary, intestinal transcriptomics technology provides a new research perspective for studying the differences in the composition and function of intestinal microbiota in different animals, which can help optimize feed formulas, regulate animal immune responses, and develop personalized nutrition plans to improve meat quality (24). Thus, understanding the multifaceted relationship between gene expression in various sites of the gut tract and its impact on the meat quality of the semitendinosus muscle is a pivotal area of research in animal science and food technology. However, there were no relevant studies reporting the changes in the transcriptome of different sites of the gastrointestinal tract, and their impact on the meat quality and transcriptome changes of the semitendinosus muscle.

Here, we sequenced 130 samples (including 118 samples from 10 sites of gut gastrointestinal tract and 12 samples from semitendinosus muscle) from sheep and goats using bulk RNA-seq. Together with meat quality (animo acid content, fatty acid content, and flavor quality) data of semitendinosus muscle. To explore candidate genes which could regulate meat quality of semitendinosus muscle in sheep and goats.

## 2 Materials and methods

### 2.1 Animals and sample preparation

In this study, six sheep (Liangshan Black Sheep) and six goats (Meigu Black Goats) from Liangshan Prefecture, Sichuan Province were used. Muscle samples from the semitendinosus of each sheep or goats were collected to examine their amino acid content, fat composition, and flavor-related metabolites. In addition, an extensive collection of gastrointestinal tract tissue samples from three sites (stomach, small intestine and large intestine) were carried out, amounting to 118 samples in total. The collected samples represented 10 essential gastrointestinal sections, including the stomach chambers (rumen, reticulum, omasum, abomasum) and the intestinal segments (duodenum, jejunum, ileum, cecum, colon, rectum). The collected samples were promptly transferred to dry ice to ensure their integrity during transportation and were ultimately stored in a −80 °C freezer for long-term preservation. Each tissue was accompanied by five to six biological replicates to enhance the reliability and robustness of the experimental data. All goats and sheep used for sample collection were of similar age (2 years) and were maintained under identical feeding environments and management conditions. During the feeding period, they exhibited comparable body weight, appearance, and health status, without any other adverse changes. All experiments were performed following the regulations set by the Institutional Animal Care and Use Committee of Xichang University (approval ID: XCU-20230708).

### 2.2 Amino acid content

Sample hydrolysis was performed to assess amino acid content. Approximately 0.1 g of each sample was precisely measured and homogenized. After adding 5 mL of 6 mol/L hydrochloric acid solution (HCl), the sample container was tightly closed and vortexed for 1 min to ensure uniform mixing. After purging the mixture with nitrogen to remove any oxygen that could interfere with the reaction, we sealed it with film and placed it in a preheated oven at 110 °C for a 24-h hydrolysis reaction. Following hydrolysis, we allowed the sample to cool to room temperature. To neutralize the solution, 5 mL of 6 mol/L sodium hydroxide (NaOH) was introduced, the tube was securely sealed, and the contents were shaken for 1 min. We then centrifuged the sample at a speed of 5,000 rpm for 10 min. We transferred half a milliliter of the supernatant into a 5 mL amber centrifuge tube, then introduced an equal volume of sodium bicarbonate solution (0.5 mol/L, pH 9.0), followed by the same volume of DNFB reagent. After sealing tightly and vortexing for 1 min, we covered the tube with film and placed it in a preheated water bath at 60 °C for 60 min, ensuring light protection to prevent photodegradation. Following the reaction, the mixture was cooled to ambient temperature, brought to a final volume of 5 mL using phosphate buffer (pH 7.0), mixed for 1 min by vortexing, and then kept in the dark for 15 min. Finally, we filtered 1 mL through a 0.22 μm membrane for analysis. For the analysis, we used an Agilent HPLC-1100 instrument equipped with a VWD detector. We employed an Agilent C18 chromatographic column (4.6 × 250 mm, 0.5 μm) with a column temperature set at 38 °C. We maintained a flow rate of 1 mL/min and an injection volume of 20 μL. The wavelength was set to 360 nm, and the mobile phase consisted of solution A (1 mol/L sodium acetate, pH 5.3) and solution B (methanol, 1:1, v/v), with separation performed under isocratic elution conditions.

### 2.3 Fatty acid content

To analyze the fatty acid content, we started with sample extraction and purification. We weighed 0.50 g of the homogenized sample and added 5 mL of extraction solvent. After rotating the mixture at high speed for 1 min, it was ultrasonically extracted in a 50 °C water bath for 90 min. Then centrifugation at 4,000 rpm, we transferred the supernatant to another tube, repeated the extraction, and combined both extracts. The sample was dried with 0.5 g of anhydrous sodium sulfate and vortexed for 30 s. Following another centrifugation at 4,000 rpm for 10 min, we transferred the supernatant to another tube and dried it with nitrogen at 50 °C to obtain the fat. Next, 5 mL of n-hexane and 3 mL of methanol–potassium hydroxide were added, followed by 60 min of vortexing, and the solution was methylated at 30 °C. Finally, we concentrated the supernatant to 0.5 mL and filtered it through a 0.22 μm membrane for analysis. For the analysis, we used an Agilent GC6890. The chromatographic column was DB-FFAP (60 × 0.25 mm) with a flame ionization detector (FID). The injection volume was 1 μL, with the detector temperature set at 280 °C and the injector temperature at 250 °C. The flow rate was maintained at 1 mL/min, with the column oven held isothermally at 180 °C. The carrier gases were supplied at flow rates of hydrogen: air: nitrogen = 40:400:40 mL/min, using a split ratio of 50:1.

### 2.4 Flavor content

To analyze the flavor content of each meat sample, we used an Agilent 8860 5977B. We placed 4 grams of each muscle sample into a 20 mL headspace bottle and added 0.8 g of NaCl. After mixing, we heated the headspace bottle containing the sample in a constant-temperature water bath at 60 °C for 25 min, allowing the volatile compounds to reach equilibrium. We then inserted the extraction needle into the headspace bottle to adsorb the volatile gas. The extraction time was set to 2,400 s using a 75 μm PDMS/DVB/CAR extraction head. We inserted the needle to a depth of 15 mm, with a coating extension length of 12 mm. The stirring speed was maintained at 300 r/min for 600 s. The analytical temperature was 270 °C, with an analysis duration of 300 s and an insertion depth of 20 mm. For the gas chromatography conditions, we used a DB-5 ms capillary column (30 m × 0.25 mm × 0.25 μm). The column temperature program began at 40 °C (2 min), increased at 6 °C/min to 160 °C (10 min hold), and then ramped at 10 °C/min to 250 °C, where it was maintained for 10 min. The carrier gas flow rate was set to 1.4 mL/min. Helium (40 mL/min) was used as the carrier gas in the mass-selective detector (MSD). The split injection ratio was 5:1, and the septum purge flow rate was 3 mL/min. The injection port temperature was maintained at 270 °C. For mass spectrometry, an electron ionization (EI) source was used at 70 eV. The ion source temperature was set to 230 °C, while the quadrupole temperature was maintained at 150 °C. The transfer line temperature was 280 °C. We operated in scanning mode (Scan/SIM) with a mass range for scanning of 35–550 and a solvent delay of 1 min.

### 2.5 RNA-seq and data analysis

The data obtained from RNA-seq were subjected to quality control using FastQC (v0.11.9), which calculated Phred quality scores, GC content, quality scores per sequence, and other metrics. The FastQC results from all samples were then integrated using MultiQC (v1.12) (3). The clean data after quality control were aligned to the reference genomes of sheep (ARS-UI_Ramb_v2.0 (25)) and goats (ARS1) (26) using STAR (v2.7.6a) (27). The expression levels of the transcripts were quantified as transcripts per million (TPM) values using Kallisto (v0.44.0) (28). Only transcripts exhibiting TPM ≥ 0.5 in at least two biological replicates were considered expressed protein-coding genes (PCGs).

All TPM values were analyzed using t-distributed Stochastic Neighbor Embedding (t-SNE) with the Rtsne (v0.17) package (29, 30). The correlation heatmap between sample pairs was obtained using the Spearman correlation coefficient. Differential expression genes (DEGs) of all samples were analyzed using DESeq.2 (v1.42.1) (31). Additionally, genes with significant differential expression were filtered by adjusting the threshold to *p* < 0.01 and |log2 fold change| > 2. All DEGs were visualized using the scRNAtoolVis (v0.0.7) software to generate volcano plots. Metascape was used to perform gene ontology enrichment analysis for each cluster to interpret the biological significance of differentially expressed genes (32, 33).

According to the average value of TPM in different tissue gene expression levels, set 0 (universal transcriptional genes) < TPM < 1 (specific genes), and then evaluate the tissue specificity of gene abundance through tau score (*τ*) (34) (the cut-off value is set as *τ* ≥ 0.75). The genes of the two sheep breeds were converted into human homologous genes using gprofiler2 (v0.2.3) (35), and then enrichment analysis was conducted. The functional annotation enrichment analysis of GO terms was performed through the clusterProfiler (v4.10.1) package (36).

To further investigate the associations between different intestinal segments and muscle phenotype-related traits, we used gastrointestinal transcriptome data to identify genes whose expression correlated with semitendinosus muscle quality traits (amino acid composition, fatty acid profiles, and flavor compounds). The top 5,000 genes with the highest median absolute deviation (variability) were selected to construct the weighted gene network analysis (WGCNA) network. Soft threshold values ranging from 1 to 20 were tested to determine the optimal threshold. The correlation matrix was transformed into an adjacency matrix, which was then converted into a topological overlap matrix (TOM). Based on the TOM, modules were classified using average linkage hierarchical clustering, with a minimum of 30 genes per module. Modules with high similarity were merged, and Pearson correlation analysis was conducted with meat quality traits. The module with the strongest positive correlation was identified as the core module. Module membership (MM) was defined as the correlation between gene expression profiles and the module eigengene.

## 3 Results

### 3.1 Meat quality of semitendinosus muscle in sheep and goats

**Figure 1:**
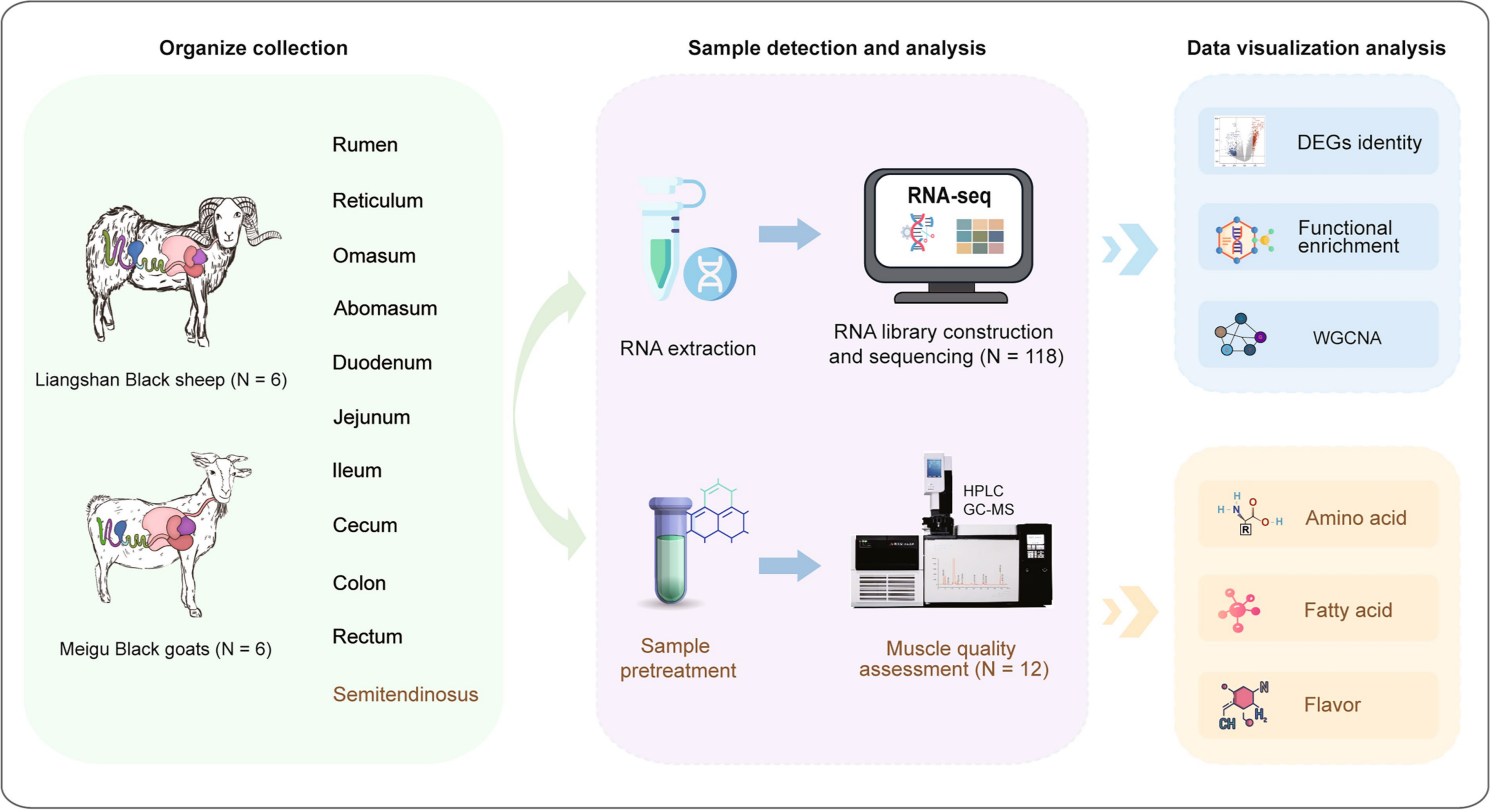
Transcriptome and meat quality analysis program diagrams of different intestinal segments and semitendinosus muscle tissues in sheep and goats.

To better understand the molecular mechanisms underlying meat quality in sheep and goats, we performed bulk RNA-seq on 130 samples, including 118 from 10 different sites of the gastrointestinal tract and 12 from the semitendinosus muscle of sheep and goats. These transcriptomic data were integrated with meat quality traits of the semitendinosus muscle, including amino acid composition, fatty acid profiles, and flavor-related indicators, to identify candidate genes potentially involved in the regulation of meat quality in sheep and goats (Figure 1).

**Figure 2:**
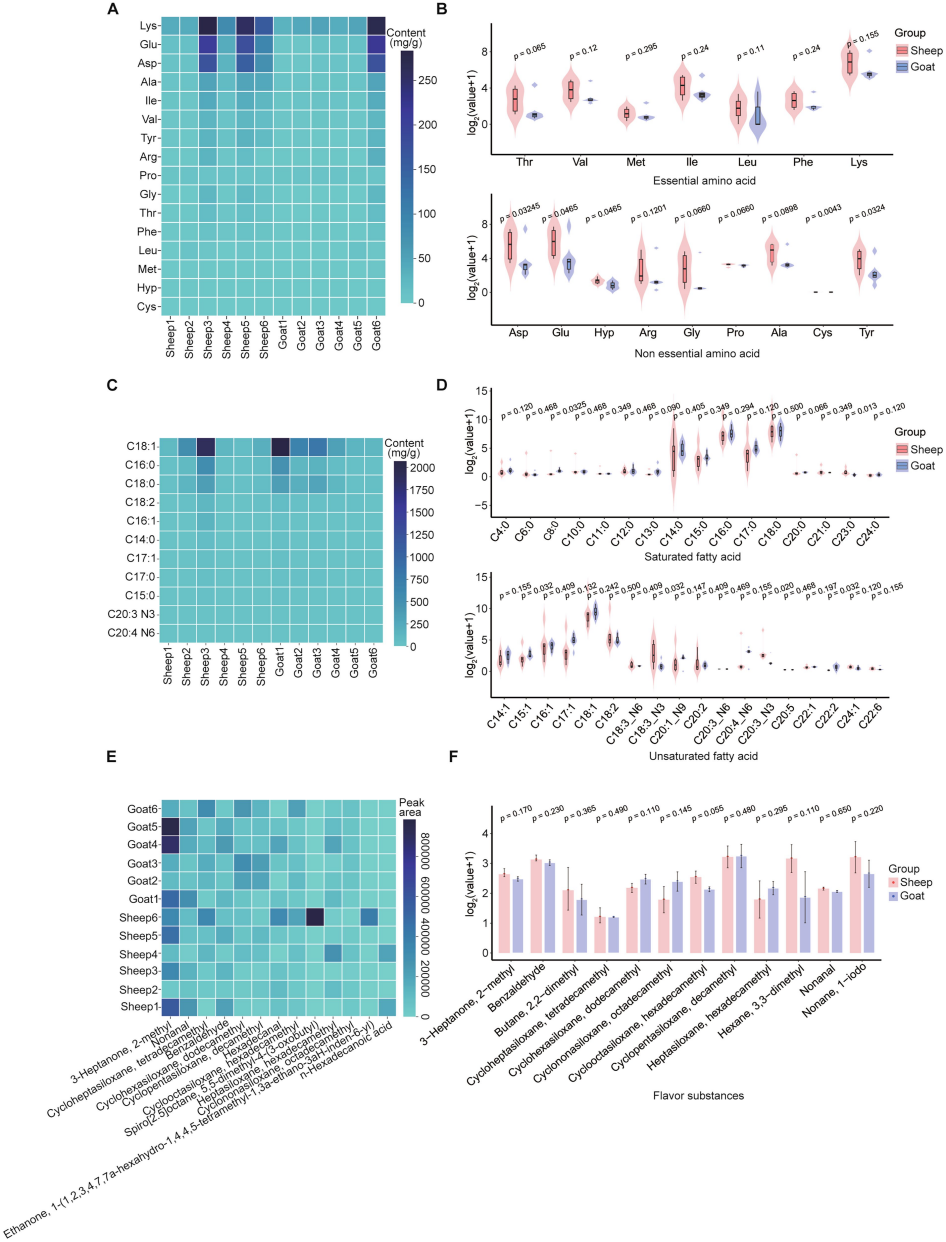
Semitendinosus muscle quality traits of sheep and goats. **(A)** Heatmap of amino acid differences in the semitendinosus muscle of each goat and sheep, showing amino acid content (mg/g) in different individuals. **(B)** Comparison of essential (upper panel) and non-essential (lower panel) amino acid contents in the semitendinosus muscle of sheep and goats (*n* = 6). Abbreviations: Thr (threonine), Val (valine), Met (methionine), Ile (isoleucine), Leu (leucine), Phe (phenylalanine), Lys (lysine), Asp (aspartic acid), Glu (glutamic acid), Hyp (hydroxyproline), Arg (L-arginine), Gly (glycine), Pro (proline), Ala (alanine), Cys (cystine), Tyr (tyrosine). **(C)** Comparison of fatty acid contents (mg/g) in the semitendinosus muscle of each goat and sheep (Top 10). **(D)** Violin plots showing the profiles of saturated (upper) and unsaturated (lower) fatty acids in the semitendinosus muscle of goats and sheep (*n* = 6). **(E)** Comparative analysis of flavor compounds in each sheep and goat (Top 10). **(F)** Bar chart showing the flavor characteristics of the semitendinosus muscle in goats and sheep (*n* = 6).

We first explored the meat quality profile of semitendinosus muscle in Liangshan sheep and Meigu goats, covering amino acid composition, fatty acid content, and flavor characteristics. The analyses revealed the amino acid and fatty acid profiles present differ in semitendinosus muscle samples from Liangshan Black Sheep and Meigu goats (Figure 2). Among the 16 amino acids (AAs), including 7 essential and 9 non-essential AAs, Liangshan Black Sheep showed higher levels of content than Meigu goats (Figures 2A,B). Notably, five non-essential amino acids—Asp, Glu, Hyp, Cys, and Tyr—showed significantly higher levels in Liangshan Black Sheep than in Meigu goats (*p* < 0.05). Similarly, of the 34 fatty acids examined, only C15:1 (*p* = 0.03), C18:3_N3 (*p* = 0.03), C20:3_N3 (*p* = 0.02), and C22:2 (*p* = 0.03) were significantly higher levels in Meigu goats than in Liangshan Black Sheep (Figures 2C,D). In addition, we examined the flavor characteristics of the semitendinosus muscle in sheep and goats. Among the detected samples, there is no significant difference of the flavor contents between sheep and goats, although there was a slightly higher in Liangshan Black Sheep for most flavor contents (Figures 2E,F).

### 3.2 Differentially expressed genes of semitendinosus muscle in sheep and goats

**Figure 3:**
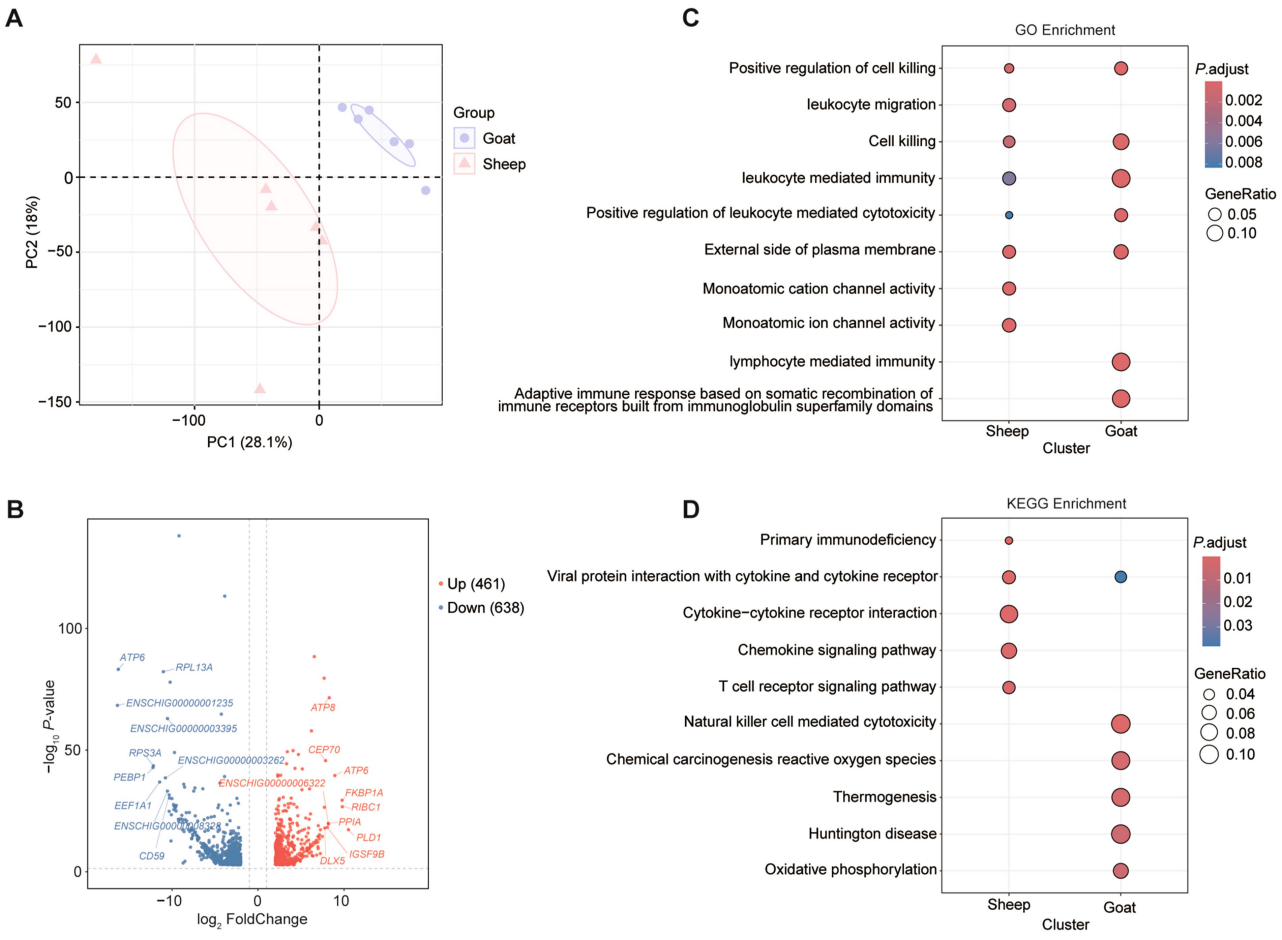
Differentially expressed genes and their functions of semitendinosus muscle in sheep and goats. **(A)** Principal component plots of sheep and goats’ semitendinosus muscle. The first dimension and second dimension are shown. **(B)** The volcano map shows the differentially expressed genes of semitendinosus muscle in sheep and goats. Red (up regulated genes) and blue (down regulated genes) dots represent the differentially expression genes. GO enrichment analysis (biological process) **(C)** and KEGG pathway enrichment **(D)** of DEGs.

A total of 881.72 Gb of high-quality sequencing data was obtained from the RNA-seq libraries of 130 sheep and goats samples, with approximately 6.78 Gb per sample (Supplementary Table 1). Analysis of 12 samples from sheep and goats revealed that the semitendinosus muscle displayed species-specific clustering. Principal component analysis (PCA) highlighted marked differences in gene expression patterns across the two breeds (Figure 3A). Analysis of differentially expressed genes (DEGs) in semitendinosus muscle identified 461 upregulated and 638 downregulated genes (Figure 3B). These DEGs were primarily associated with GO enrichment terms including “Monoatomic cation channel activity,” “Leukocyte migration,” and “Monoatomic ion channel activity” in sheep; “Lymphocyte mediated immunity” and “Adaptive immune response based on somatic recombination of immune receptors built from immunoglobulin superfamily domains” in goats (Figure 3C), as well as KEGG pathways such as “Cytokine-cytokine receptor interaction,” “Chemokine signaling pathway,” and “T cell receptor signaling pathway” for sheep and “Natural killer cell mediated cytotoxicity,” “Chemical carcinogenesis reactive oxygen species,” “Thermogenesis,” and “Oxidative phosphorylation” for goats (Figure 3D).

### 3.3 Differentially expressed genes of 10 gastrointestinal tract tissues

**Figure 4:**
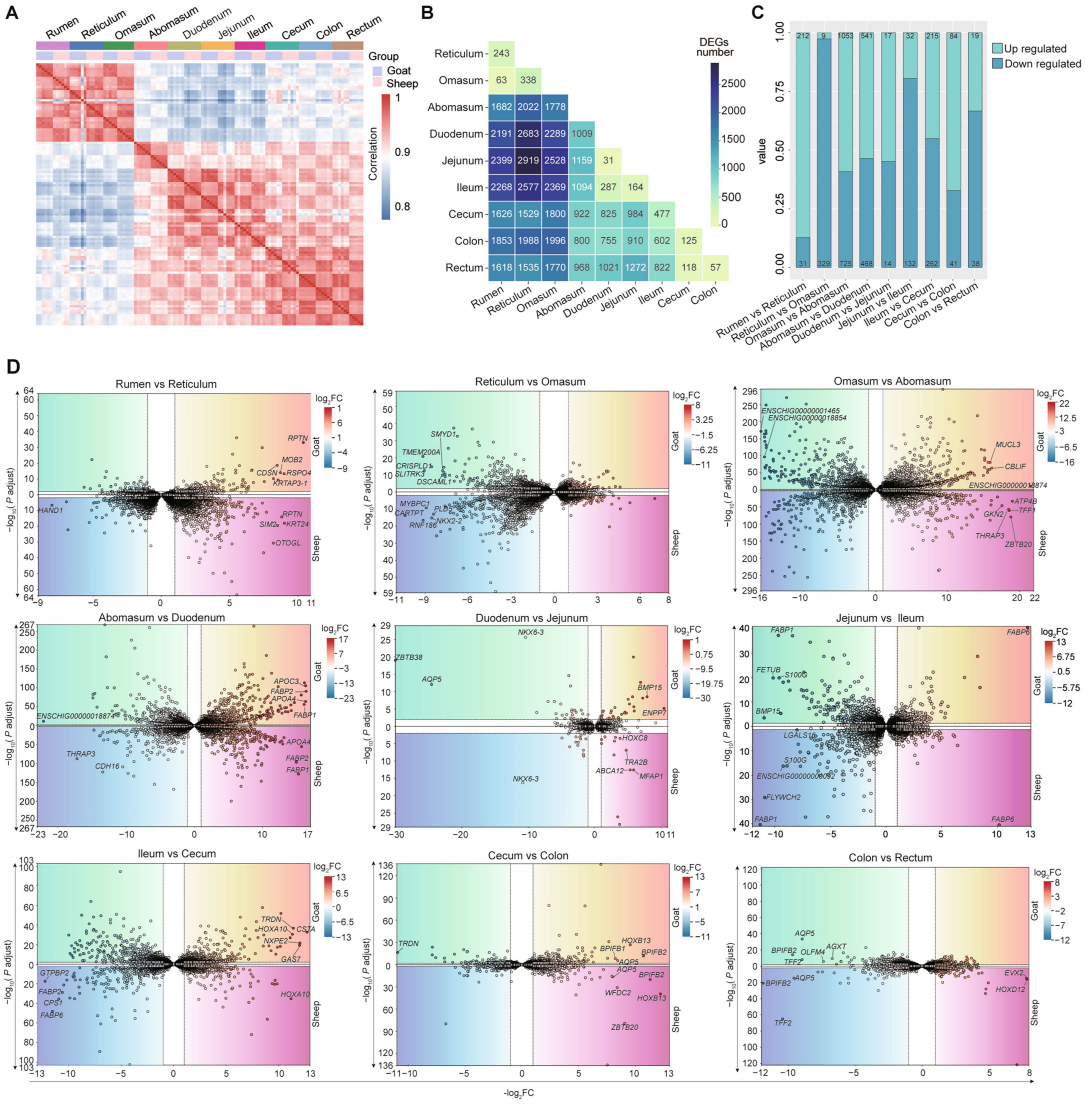
Differential transcriptomic analysis of 10 gastrointestinal tract tissues. **(A)** The spearman correlation heatmap shows the correlations among RNA-seq samples from different intestinal segments of the gastrointestinal tracts of 10 goats and sheep. Gray-blue (low correlation), red (high correlation). **(B)** Number of differentially expressed genes (DEGs) among 10 intestinal tissues. **(C)** The number and proportion of up-regulated (cyan) and down-regulated (blue) genes in the anterior and posterior intestinal segments of goats and sheep (9 comparisons in total). **(D)** Volcano plots of DEGs between anterior and posterior intestinal gastrointestinal tract sites (9 comparisons in total) in both goats (upper panels) and sheep (lower panels).

Next, we examined the expression profiles of 10 gastrointestinal tract tissues in adult sheep and goats. To characterize the transcriptome-wide profile (the overall expression patterns of all detected genes), we performed Spearman correlation and DEGs analyses. Strongest correlations were observed across the six replicate samples collected from different sites (Figure 4A). Rumen, reticulum and omasum showed a similar expression pattern among them and differed from other 7 sites (Figure 4A). Then, we performed differential expression analyses between each pair of gastrointestinal sites and assessed the number of DEGs showing significant changes in each comparison, jejunum and duodenum possessed the least number of DEGs (only 31 DEGs were detected); the difference of gene expression between jejunum and reticulum showed the greatest number of DEGs (2,919 DEGs were detected) (Figure 4B). The difference in gene expression between sheep and goats became smaller as they moved to the last three sites (cecum, colon, and rectum). As there are only 125 and 57 DEGs for Cecum VS Colon and Colon VS Rectum comparisons (Figure 4C). The greatest number of DEGs between the two adjacent sites was Omasum VS Abomasum (1,778). Similar patterns were also found when only analyzed the differential expression among 10 gastrointestinal tract tissues in sheep and goats, respectively, (Supplementary Figure 1). Volcano plots revealed distinct transcriptional signatures between adjacent gastrointestinal segments in these small ruminants (Figure 4D). The transition from rumen to reticulum was marked by strong upregulation of *RPTN*, *MOB2*, and *OTOGL*. Reticulum-to-omasum differences involved reproductive- and cytoskeleton-related genes such as *PLD5*, *SMYD1*, and *CARTPT*. In the omasum–abomasum comparison, secretory and signaling genes (*MUCL3*, *CBLIF*, *GKN2*) were differentially expressed. Striking changes from abomasum to duodenum included lipid absorption genes (*APOC3*, *APOA4*, *FABP1*, *FABP2*). Duodenum-jejunum and jejunum-ileum transitions involved nutrient transporters and binding proteins (*BMP15*, *ABCA12*, *FABP1*, *S100G*, *HOXC8*). The ileum-cecum boundary displayed shifts in metabolic and immune-related genes (*TRDN*, *NXPE2*, *CSTA*, *HOXA10*), while cecum-colon differences included *BPIFB2*, *WFDC2*, and *AQP5*. Minimal transcriptional changes were observed between colon and rectum, mainly involving *AQP5* and *BPIFB2* (Figure 4D).

**Figure 5:**
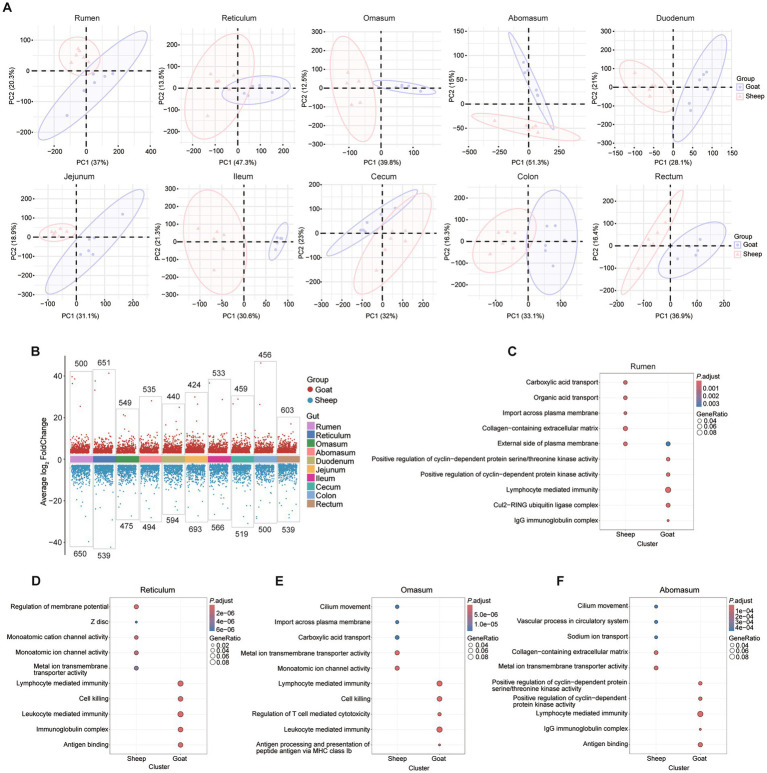
Transcriptomic differences of 10 gastrointestinal tract segments in goats and sheep. **(A)** PCA plots showing that samples cluster in each segment between sheep and goats. **(B)** Volcano plots of differentially expressed genes (DEGs) between goats and sheep across the 10 segments. **(C–F)** Representative GO enrichment of DEGs in rumen, reticulum, omasum, and abomasum.

For comparisons at the same sites between two breeds, we conducted the PCA analysis of each site between the sheep and goats (Figure 5A). The volcano plots of DEGs between goats and sheep across the 10 gut segments revealing both shared and segment-specific transcriptional differences (Figure 5B). Representative GO enrichment results for DEGs in selected gut regions (rumen, reticulum, omasum, and abomasum) revealed their different functions. These DEGs were mainly involve immune-related processes—such as antigen binding, immunoglobulin complex formation, lymphocyte-mediated immunity, and antigen processing—as well as ion transport (especially metal and cation channels), extracellular matrix components, and cilium movement (Figures 5C–F). GO enrichment results for the remaining gut regions (duodenum, jejunum, ileum, cecum, colon, and rectum) also show strong enrichment for adaptive immune responses involving immunoglobulin domains, antigen binding, and lymphocyte-mediated immunity, alongside region-specific processes such as lymphocyte mediated immunity (jejunum), muscle contraction (ileum), monoatomic ion channel activity (lleum, cecum, rectum), and apical plasma membrane organization (colon, rectum) (Supplementary Figure 2). For the KEGG analysis, the results revealed segment-specific functional divergence between sheep and goats: proximal segments (rumen, reticulum, omasum) are enriched in immune and metabolic pathways, mid-gut segments (duodenum, jejunum, ileum) show strong signatures of lipid handling and oxidative metabolism, while distal segments (cecum, colon, rectum) exhibit pathways related to detoxification, cardiomyopathy-related signaling and folate metabolism (Supplementary Figure 3).

### 3.4 Landscape of gene dynamic expression in 10 gastrointestinal tract segments

**Figure 6:**
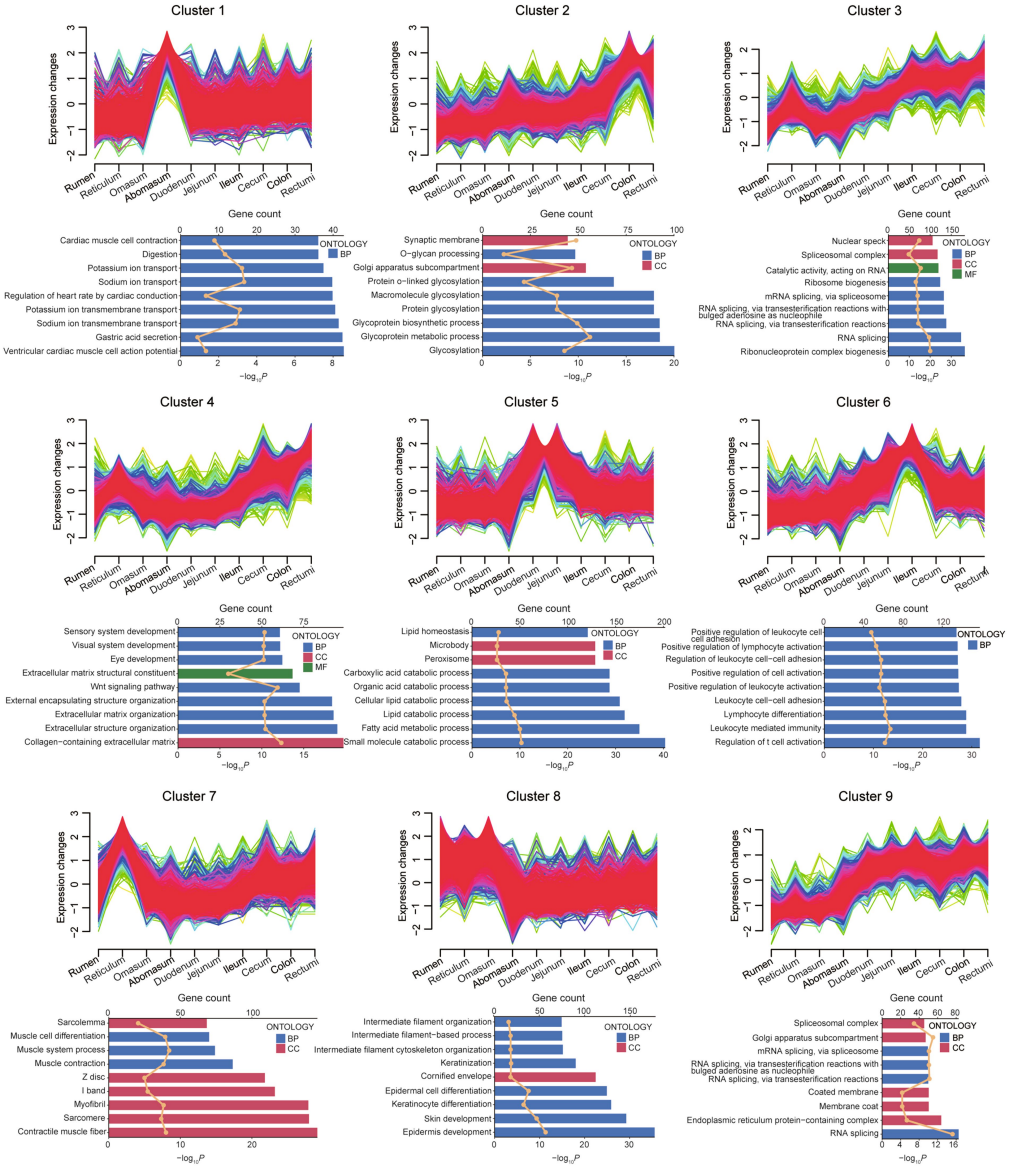
Dynamic gene expression landscapes of across 10 different gastrointestinal tract segments sites. Upper panels of each cluster depict the fuzzy c-means clustering results for 9 unique gene expression profiles. The x-axis indicates 10 distinct intestinal sites, while the y-axis shows the standardized TPM values of genes. Lower panels were GO terms for each cluster. BP: Biological Processes, CC: Cellular Component. The upper axis corresponds to the line plot and represents the number of genes, and the lower axis corresponds to the bar plot and represents the *p* value.

To investigate the sequential transcriptome dynamics across 10 gastrointestinal sites in small ruminants, we utilized the fuzzy c-means algorithm for clustering gene expression data. Gene expression patterns across 10 consecutive intestinal regions from the rumen to the rectum, organized into nine clusters (Cluster 1–9) (Figure 6). Each cluster exhibits distinct expression trends, with some showing gradual increases (Cluster 2, 3, 4, and 9) or first rise then descend (Cluster 1, 5, and 6) along the gastrointestinal tract. Functional enrichment analysis reveals diverse biological processes associated with these clusters. For instance, Cluster 1 is linked to cardiac muscle activity and ion transport, Cluster 2 to glycosylation and glycoprotein metabolism, and Cluster 3 to RNA splicing and ribonucleoprotein complex biogenesis. Clusters 4–6 highlight extracellular matrix organization, lipid catabolism, and immune-related processes, respectively. Clusters 7–9 are associated with muscle contraction, epidermal development, and endoplasmic reticulum functions. These findings suggest region-specific gene expression dynamics that correlate with the functional specialization of different intestinal segments. The clustering analysis across 10 gastrointestinal regions, using combined data from sheep and goats (Figure 6), revealed consistent expression patterns and functional enrichments along the tract from rumen to rectum. Separate analyses for sheep (Supplementary Figure 4) and goats (Supplementary Figure 5) showed highly similar trends, indicating that gastrointestinal functional organization is largely conserved among these small ruminants.

### 3.5 Key sites in the process of digestion

**Figure 7:**
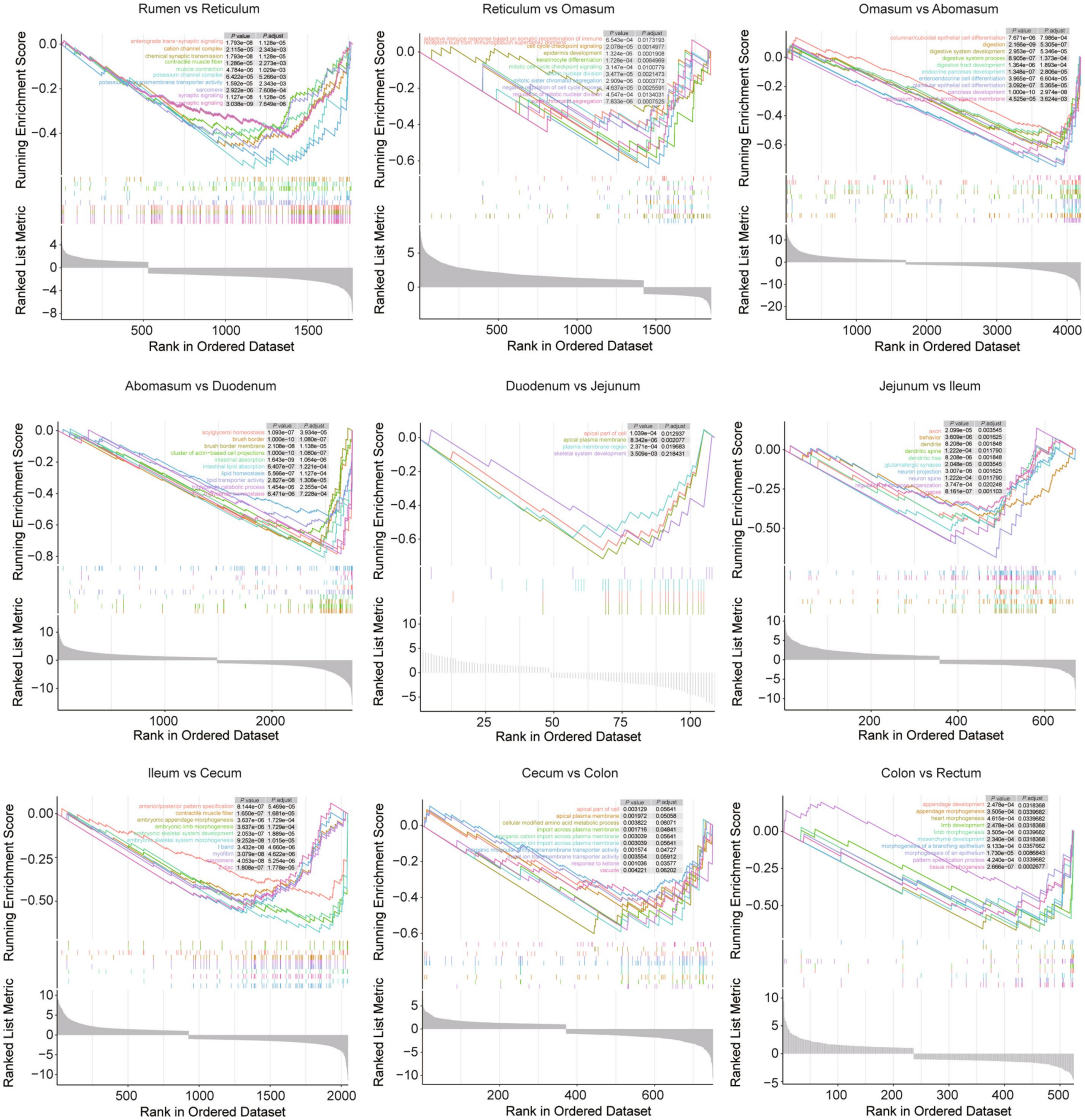
The GSEA plot presents the top 10 enriched terms for comparisons between conjoint gastrointestinal tract sites, with different colors representing distinct terms. The panel in the upper right corner lists the adjusted *p* values for each term.

The gastrointestinal tract plays a crucial role in digesting food and absorbing nutrients, with each intestinal region performing distinct functions. To explore the molecular basis of these functional differences, combined analyses were conducted across various gastrointestinal tract sites (Figure 7). Gene-set enrichment analysis (GSEA) across the nine consecutive comparisons revealed that the strongest functional shifts occur at three transition zones. Such as, from rumen to reticulum: anterograde trans−synaptic signaling, cation channel complex, chemical synaptic transmission, contractile muscle fiber, and muscle contraction dominate the leading edge. From omasum to abomasum: a sharp surge in “digestive/absorptive” signatures-accompanied by enteroendocrine cell differentiation, and glandular epithelial cell differentiation pathways. From abomasum to duodenum, the switch from gastric to small-intestinal function is marked by intestinal lipid absorption, acylglycerol homeostasis, and lipid transporter activity. After the jejunum, the magnitude of enrichment gradually declines; ileum to cecum and cecum to colon show only modest changes, mainly involving cellular modified amino acid metabolic process, contractile muscle fiber and morphogenesis of a branching epithelium, indicating functional convergence in the hindgut. When compared in sheep and goats separately, sheep show the strongest enrichment for cholesterol metabolism in comparison between abomasum and duodenum (Supplementary Figure 6). Goats display an earlier metabolic switch: from rumen to reticulum already shows significant enrichment for glycolysis/gluconeogenesis and ferroptosis (Supplementary Figure 7).

### 3.6 Network analysis of different gastrointestinal tract sites based on weighted gene co-expression network analysis (WGCNA)

**Figure 8:**
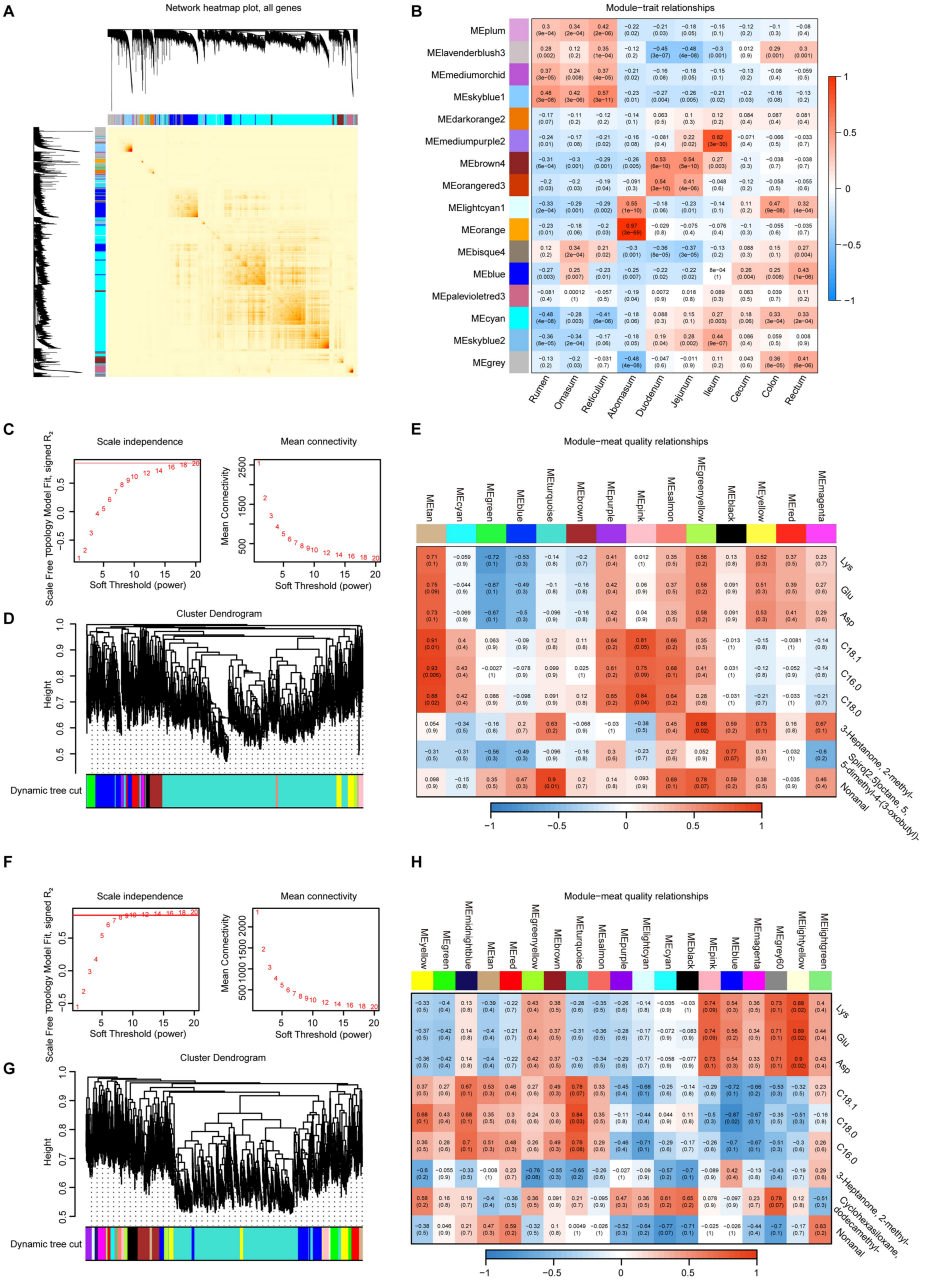
WGCNA of gastrointestinal transcriptomes and their associations with meat quality traits in sheep and goats. **(A)** Heatmap of the topological overlap matrix for all genes, where darker blocks along the diagonal represent 16 distinct modules. **(B)** Heatmap illustrates the correlations between modules and gastrointestinal tract sites. Mean connectivity for scale independence and soft threshold (*β*) for sheep **(C)** and goats **(F)** data. Gene clustering dendrograms for sheep **(D)** and goat data **(G)**. Heatmaps of the correlation between module eigengenes and meat quality data for sheep **(E)** and goats **(H)**.

Furthermore, we employed WGCNA to explore the gene network in various intestinal regions, identifying a total of 16 distinct modules (Figure 8A). The spearman correlation coefficients were determined for each module in relation to each sample. The correlations between gene expression in the mediumorchid and skyblue1 modules and the various intestinal sites tended to decrease along the digestive system, from the rumen to the rectum. On the other side, palevioletred3, cyan, skyblue2 and gray showed increase strends. The hierarchical clustering heatmap revealed that the abomasum exhibited the most distinct clustering among the ruminant gastrointestinal regions (Figure 8B).

To further refine our analysis, we performed weighted gene network analysis (WGCNA) on sheep and goats. In sheep, network analysis identified multiple co-expression modules that exhibited distinct associations with meat quality traits (Figures 8C–E). For example, the tan and pink modules were positively correlated with the top three fatty acid content; the green-yellow module was significantly positively correlated with 3-heptanone, 2-methyl-; and the turquoise module was significantly positively correlated with nonanal. This suggests that sheep-specific gastrointestinal gene expression programs may influence fatty acid metabolism and the biosynthesis of flavor-related metabolites in muscle. Similarly, in goats, distinct modules were associated with meat quality traits (Figures 8F–H). The light-yellow module was positively correlated with amino acid content (e.g., Lys, Glu, and Asp); C18:0 was positively correlated with the turquoise module and negatively correlated with the blue module, indicating a higher degree of amino acid enrichment integration in goats compared to sheep. These results indicate that while both sheep and goats exhibit modular organization of gastrointestinal gene expression, module-trait relationships differ between species. This provides new insights into the species-specific regulatory mechanisms by which intestinal transcriptional networks shape the amino acid, fatty acid, and flavor traits of semitendinosus muscle.

## 4 Discussion

### 4.1 Segment-specific gene programs and functional zonation of the gut

Different regions of the gastrointestinal tract exhibit distinct physiological roles, including digestion, nutrient absorption, immune modulation, and microbial interaction (14, 37). These regional functions are closely associated with spatial differences in gene expression profiles, which reflect local adaptation to specific physiological tasks. Such region-specific expression contributes to shaping important host phenotypes, including metabolic efficiency, growth traits, and immune responses (38, 39). Gene expression within these gut segments is known to influence the digestive processes, nutrient utilization, and the overall metabolic activity of the organism (40). Moreover, the gut microbiota, which also contributes to the gene expression profile in the gut, has been increasingly recognized for its impact on host physiology and metabolism, including potential effects on meat quality attributes (14, 37). Nevertheless, the distinct functions of different gastrointestinal tract (GIT) segments lead to variations in gut microbial communities across regions and functions (41). Over the past few decades, the economic significance of livestock meat quality has spurred extensive research aimed at boosting production, hastening growth rates, and enhancing meat quality (42). The application of multi-omics strategies significantly benefits the analysis of complex traits (15, 43–45). Researchers identified 463 transcripts with consistent expression patterns in the semitendinosus of beef-breed bulls compared to dairy-breed bulls; the upregulated genes in beef breeds were mainly associated with protein production and degradation, which may underlie their enhanced muscle growth (46). Therefore, understanding transcriptomic variation along the gut provides critical insight into the molecular basis of complex traits in livestock and other species. This study generated the first segment-resolved transcriptomic atlas spanning the entire gastrointestinal (GI) tract together with the semitendinosus muscle in sheep (Liangshan Black) and goats (Meigu Black) and linked region-specific gene expression to meat-quality traits.

The rumen-reticulum transition is dominated by ferroptosis, pyruvate metabolism and natural-killer-cell-mediated cytotoxicity (Supplementary Figure 3). Ferroptosis genes can regulate rumen epithelial turnover and subsequently modulate SCFA transporter expression (47). The SCFA-GPR41/43 axis is established as a systemic metabolic signal that up-regulates muscle oxidative genes (48), here we found muscle genes such as *SMYD1* and *MYBPC1* were detected as DEGs. These results indicated that the initial sections of the forestomach, namely the rumen and reticulum, exhibited strong muscular contractions to support microbial fermentation and energy generation (49).

The omasum-abomasum switch is marked by a surge in “digestive/absorptive” signatures—cholesterol biosynthesis, PPAR signaling and glandular epithelial differentiation (Figure 7). Key genes (*APOC3*, *APOA4*, *GNK2* and *FABP* members) encode intestinal lipid-binding proteins that shuttle fatty acids to portal circulation. In *FABP4*/*FABP5* double-knockout mice, loss of both lipid chaperones severely blunts trans-endothelial fatty-acid transport into red skeletal muscle and the heart, forcing these tissues to switch to glucose as their primary fuel—even under fasting conditions—when fatty-acid supply normally predominates (50), mirroring the higher C18:3N3 phenotype we observed in sheep whose abomasum abundantly expresses *FABP* genes. *GKN2*-knockout mice exhibit thinner mucus and altered SCFA profiles that reduce muscle glutamate levels and loss of *GKN2* drives premalignant gastric inflammation and tumor progression (51). Thus, abomasum *GKN2* may indirectly control umami-related amino acids in the semitendinosus muscle.

Mid-gut segments are enriched for PPAR and ABC transporter pathways (Supplementary Figure 3). *ABCA12* and *S100G*, up-regulated in jejunum, mediate cholesterol efflux and calcium absorption—processes coupled to muscle fiber-type switching via calcineurin–NFATc1 signaling (52). Cecal *AQP5* expression is linked to water transport capacity, and the protein influences downstream molecules that participate in multiple cellular activities (53), suggesting an analogous role in ovine/caprine meat.

Hind-gut segments display detoxification (cytochrome P450), folate biosynthesis and oxidative phosphorylation pathways. The cecal expression of *WFDC2* is associated with mucin O-glycan biosynthesis, and by sustaining epithelial tight junction integrity, *WFDC2* limits bacterial infiltration and mitigates mucosal inflammation (54); *BPIFB2* (also known as *LPLUNC2*) belongs to the BPI-fold-containing family B and is part of the lipid-transfer/lipopolysaccharide-binding protein (LT/LBP) super-gene family. *BPIFB2* secreted into the airway and intestinal lumen where it modulates tight-junction proteins and limits bacterial translocation (55).

### 4.2 Species-specific divergences

For the meat quality difference, in semitendinosus muscle, sheep had higher overall amino acid content, especially Asp, Glu, Hyp, Cys, and Tyr, which play roles in umami taste (Asp, Glu) and connective tissue integrity (Hyp). This suggests sheep may naturally produce meat richer in flavor precursors and structural proteins. Protein-derived flavor constitutes a distinct gustatory dimension, separate from the sensations elicited by sweetness, saltiness, or the rich notes of cheese and vegetables exemplified by tomato and mushroom types (56). The umami taste is enhanced by the combined action of five nucleotides and glutamate (57). Primary umami triggers are monosodium L-glutamate along with inosine 5′-monophosphate. Amino acids other than glutamate, particularly aspartate and theanine—also further contribute to this savory profile (56). Goats meat had slightly higher levels of C15:1, C18:3N3 (α-linolenic acid), C20:3N3, and C22:2, reflecting possible differences in lipid absorption or rumen biohydrogenation capacity (58). No significant differences in volatile profiles were found, but sheep trended higher for most compounds. The results show strong regional differentiation in gene expression, consistent with prior pig GI atlases (38).

For the gut segments mRNA expression difference, goats showed transcriptional signatures in rumen epithelium suggesting stronger oxidative phosphorylation and n-3 PUFA retention, matching the higher α-linolenic acid observed in meat. Sheep displayed higher amino acid metabolism gene expression in small intestine, consistent with higher muscle amino acid content. These patterns may reflect evolutionary adaptation—goats often graze on more browse-type diets rich in plant secondary metabolites, influencing lipid metabolism gene regulation (59). Sheep show pronounced cholesterol-handling signatures at the abomasum–duodenum junction (Supplementary Figure 6), consistent with higher muscle cholesterol content reported in hill-grazed lambs (60). Goats, by contrast, display earlier activation of glycolysis/ferroptosis genes in the rumen-reticulum (Supplementary Figure 7), aligning with their leaner carcass phenotype and elevated poly-unsaturated fatty acids (Figure 2D). These gene-level differences underpin breed-specific meat-quality attributes.

Although bulk RNA-seq provided a tract-wide overview, it cannot resolve cell-type heterogeneity. To explore the formation of animal traits, future studies will integrate rumen and cecal metagenomes with metabolomic profiles, which will be critical for validating causal links among microbiota, host gene expression, and meat-quality phenotypes. In summary, our segment-centric transcriptomic approach delineates a “gut–muscle axis” in small ruminants and identifies region-specific genes (e.g., *RPTN*, *GKN2*, *APOC3*, and *AQP5*) and pathways (muscle contraction, O-glycan processing, and lipid homeostasis) that may be influenced by nutritional or microbial interventions to tailor semitendinosus muscle meat quality (61).

## 5 Conclusion

This study provides the first segment-resolved transcriptomic atlas of the gastrointestinal tract in sheep and goats linked directly to meat quality traits. Our findings reveal three key transition zones along the GI tract that correspond to major functional and metabolic shifts impacting semitendinosus muscle composition. The results highlight a gut–muscle axis whereby specific GI tract gene expression patterns influence fatty acid profiles, amino acid content, and flavor precursors in skeletal muscle. Species-specific transcriptional and phenotypic differences suggest distinct metabolic adaptations in sheep and goats, offering molecular targets for selective breeding or dietary modulation. These insights advance our understanding of small ruminant digestive physiology and provide a genetic basis for improving meat quality.

## Data Availability

The datasets presented in this study can be found in online repositories. NCBI repository, https://www.ncbi.nlm.nih.gov/bioproject/PRJNA1067820/.
