# Supplementary material for: The expression of genes in different sites of gut tract regulates the meat quality of semitendinosus muscle in sheep and goats
Source: Front Vet Sci. 2025 Oct 29;12:1687258. doi: 10.3389/fvets.2025.1687258 (PMC12605001; doi:10.3389/fvets.2025.1687258)
Supplement: Supplementary file 1 [file Data_Sheet_1.docx]

Supplementary Material

## Supplementary Figures

**Supplementary Figure 1.** Differential transcriptomic analysis of 10 gastrointestinal tract tissues in sheep and goats. Number of differentially expressed genes (DEGs) among 10 intestinal tissues in sheep **(A)** and goats **(B)**. **(C)** Bar plot showing the number and proportion of upregulated (cyan) and downregulated (blue) genes in anterior vs. posterior intestinal segments (9 comparisons in total) in sheep **(C)** and goats **(D)** respectively.

**Supplementary Figure 2.** GO enrichment for duodenum **(A)**, jejunum **(B)**, ileum **(C)**, cecum **(D)**, colon **(E)**, and rectum **(F)** respectively.

**Supplementary Figure 3.** KEGG pathway enrichment for 10 gastrointestinal tract segments in goats and sheep.

**Supplementary Figure 4.** Dynamic gene expression landscapes of across ten different gastrointestinal tract segments sites in sheep. Upper panels of each cluster were clustering of fuzzy c-means of 9 distinct patterns of gene expressions. The x-axis represents 10 different intestinal sites, and the y-axis represents TPM values of genes after standardization. Lower panels were GO terms for each cluster. BP: Biological Processes, CC: Cellular Component. The upper axis corresponds to the line plot and represents the number of genes, and the lower axis corresponds to the bar plot and represents the *P* value.

**Supplementary Figure 5.** Dynamic gene expression landscapes of across ten different gastrointestinal tract segments sites in goats. Upper panels of each cluster were clustering of fuzzy c-means of 9 distinct patterns of gene expressions. The x-axis represents 10 different intestinal sites, and the y-axis represents TPM values of genes after standardization. Lower panels were GO terms for each cluster. BP: Biological Processes, CC: Cellular Component. The upper axis corresponds to the line plot and represents the number of genes, and the lower axis corresponds to the bar plot and represents the *P* value.

**Supplementary Figure 6.** The GSEA plot presents the top 10 enriched terms for comparisons between conjoint gastrointestinal tract sites in sheep, with different colors representing distinct terms.

**Supplementary Figure 7.** The GSEA plot presents the top 10 enriched terms for comparisons between conjoint gastrointestinal tract sites in goats, with different colors representing distinct terms.
